# Analysis of incidence of postoperative wound infection in closed fractures treated by surgical fixation – A prospective study

**DOI:** 10.1016/j.amsu.2021.103029

**Published:** 2021-11-09

**Authors:** Niranj Ganeshan Radhamony, Radhu Raj, Sivakumar Raju

**Affiliations:** a Royal Stoke University Hospitals, Stoke on Trent, UK; b Department of Prosthodontics, Amrita Institute of Medical Sciences, Kochi, Kerala, India; c PIMS Multi Speciality Hospital, Madurai, Tamilnadu, India

**Keywords:** Wound infection, Surgical site infection, Orthopaedic device related infection, Orthopaedic surgery, Operative duration, Diabetes

## Abstract

**Background:**

In orthopaedic surgery where metallic implants are used, surgical site infection (SSI) is a significant complication as it increases the postoperative morbidity and mortality, prolongs hospital stay, and increases the hospital costs. Therefore, understanding the incidence of SSI in various healthcare settings would help us analyse the contributing factors and improve healthcare. Since the rates of SSI in various orthopaedic settings in India are lacking, this study aimed at analysing them and various associated factors in a tertiary medical institute in India.

**Materials and methods:**

This study was a prospective cohort study carried out on 100 orthopaedic patients who underwent surgical fixation for closed fractures, with a follow-up period of one year. The incidence of SSI and the factors associated with them were analysed.

**Results:**

The overall incidence of SSI in our study was 5% (5 cases). The maximum incidence was seen in the age group 51–60 years (40% of the infections). Regarding the duration of preoperative hospital stay, the incidence of SSI was 6.85% when the patients stayed for more than 10 days, whereas the incidence was only 1.66% when the patients stayed for less than or equal to 10 days. Among the diabetics, the incidence of infection was found to be 7.69% (1 out of 13 diabetics). Regarding the operative duration, while cases which took more than 1.5 hours of operative duration had the highest infection rate (60% of the infections), none of the cases which took less than an hour to operate got infected. Among the infective organisms isolated, 60% cases had *Staphylococcus aureus*, 20% had *Proteus vulgaris* and another 20% had *Klebsiella pneumoniae* infection.

**Conclusions:**

Age greater than 50 years, a prolonged preoperative hospital stay more than 10 days, presence of diabetes, a prolonged surgical procedure more than 1.5 hours, and were found to be at a higher risk of SSI in our study.

## Highlights

• Surgical site infection (SSI) in orthopaedic surgery can lead to chronic infection or osteomyelitis.
• Specific data about SSI in orthopaedics in India are lacking.
• Our study highlighted the SSI related factors in orthopaedics in India.
• Higher age, longer preoperative hospital stay, prolonged surgical procedure and diabetes were found associated.

## 1 Introduction

Surgical site infection (SSI), categorized under the broad term nosocomial infection, poses a significant problem to the healthcare system because it increases the chance of postoperative morbidity and mortality, prolongs hospital stay, and increases healthcare costs [[1], [2], [3], [4]]. It has been shown that wound infection increases hospital stay by three to four times, and reduces the survival chance until discharge by up to four times [[5], [6], [7], [8]]. Again, such an increased stay in the hospital blocks beds and may triple or quadruple the associated costs [9,10]. These infections are usually caused by exogenous and endogenous microorganisms, mostly aerobic and anaerobic bacteria that contaminate the operative wound during or after surgery. Moreover, SSI poses a greater threat to orthopaedic surgeries than various others because of the usage of metallic implants that harbour the pathogens thereby making the elimination of infection extremely difficult [11,12]. Hence, the term Orthopaedic Device Related Infection (ODRI) has been introduced, and various studies have shown that a duration of at least one year needs to be elapsed before ruling out SSI when implants are used [13].

The incidence of SSI varies between 1% in certain hospital settings in Europe and the USA to a very high value of 20% in parts of Asia and Sub-Saharan Africa [14]_._ However, sufficient data about the incidence of SSI in specific orthopaedic settings in India is lacking. Since the majority of treatment in the Indian healthcare system is being provided by government institutions, it is crucial to analyse their incidence and contributing risk factors in a government institutional setup. Among orthopaedic surgeries, fixation of fractures contributes to a larger proportion of overall cases. Therefore, we aimed at analysing the incidence of SSI in closed fractures treated by surgical fixation in a tertiary medical centre in India.

## 2 Materials and methods

This prospective observational cohort study was conducted from July 2015 to December 2016, after obtaining Institutional Ethics Committee approval (IEC- GRH 005420), and the work has been reported in line with the STROCSS criteria [15].

We included a total of 100 patients, who underwent surgical fixation for closed fractures on an elective basis between July 2015 and December 2015, and all the participants consented for the study. We included all types of closed fractures in patients of age 18 years and above treated by surgical fixation. The exclusion criteria were immunocompromised patients, open fractures, joint replacement procedures, arthroscopic procedures, soft tissue procedures and spine surgeries.

The routine infection control measures in this hospital included shaving the hair of the involved limb and surgical site about 6–12 hours before the procedure. All the patients received an antibiotic prophylaxis of 1g of inj Ceftriaxone and 500 mg of inj Amikacin, after confirming the renal parameters, 45 minutes before the surgical procedure. For patients with penicillin allergy, an alternative medication was given as per microbiology advice. All the aseptic precautions like maintaining operation theatre sterility, performing surgical hand scrubbing for 3–5 minutes, and the usage of autoclaved gowns, drapes, sterile gloves, instruments and implants were followed during the operative procedure [16]. The operation theatres had air conditioning but were not a laminar flow system. The theatre staff wore a surgical scrub before entering the theatre complex and donned in sterile autoclaved surgical gowns. The hand scrub was performed for 3–5 minutes using 5% povidone iodine scrub solution.

The operative site was painted with 5% *povidone iodine* before draping, the incision site was painted using *surgical spirit* before making the incision. All the cases were operated by Assistant Professors, Associate Professors or Professors of the institution. The hospital surgical aseptic protocol was followed irrespective of surgeon bias. Adequate haemostasis was maintained in all the cases and drains used in certain cases, as per the surgeon's preference. Skin closure was done with 2-0 silk sutures, and the wounds were covered with adhesive dressings.

Postoperatively, intravenous antibiotics were continued until the second postoperative day. The patients were observed for signs of infection (SSI) like redness, swelling, local raise of temperature, pus discharge, fever, regional lymphadenopathy and elevated inflammatory blood parameters like C reactive protein (CRP), white blood count (WBC) and erythrocyte sedimentation rate (ESR) on the 2nd, 6th and 12th post-operative day, and at the end of 3 months, 6 months and 1 year. The infected patients were managed as per the hospital's infection control protocol which included monitoring blood parameters, performing wound swab culture and sensitivity, initiating appropriate antibiotics and wound debridement and washout whenever necessary.

## 3 Results

Out of the total 100 patients, 5 patients developed SSI. The variable-wise analysis indicates that the age, duration of surgery, preoperative hospital stay and presence of diabetes could be the contributing factors to the development of SSI.

**Age and Sex:** Though statistically not significant, two cases showed signs of SSI in the age group 51–60 years (10% cases in this group). The other cases were distributed as shown in Table 1. Sex-wise distribution of the patients showed the development of SSI in 4 out of 81 male patients (4.93%) and 1 out of 19 female patients (5.26%).

**Table 1:** Age wise distribution of the patients who developed SSI.

Age group (years)	Total No of patients	No of patients with SSI	Percentage of SSI in that age group
18 to 30	22	1	4.54
31 to 40	16	0	0
41 to 50	28	1	3.57
51 to 60	20	2	10
Above 60	14	1	7.14

**Pre-operative hospital stay:**Table 2 depicts the distribution of SSI cases according to the duration of preoperative hospital stay, classified as 5- day increments. It is notable that the incidence of SSI was 6.85% when the patients stayed for more than 10 days whereas, the incidence was only 1.66% when the patients stayed for less than or equal to 10 days before the operation. None of the patients developed postoperative infection in the period less than 5 days.

**Table 2:** Incidence of SSI in relation to preoperative hospital stay.

Duration of preoperative hospital stay (days)	Total No. of patients operated	No. of patients infected	Percentage
<5	8	0	0
6–10	30	1	3.33
11–15	36	3	8.33
Above 15	26	1	3.84

**Diabetes and SSI:**Table 3 shows the incidence of infection in diabetics. It is seen that 1 patient out of the 13 diabetics developed SSI in the age group 41–50 years. All the diabetic patients were rendered euglycemic before considering surgical intervention.

**Table 3:** Incidence of SSI among diabetic patients.

Age group (years)	No of diabetic patients operated	No of infections in diabetic patients	Percentage
18 to 30	0	0	0
31 to 40	0	0	0
41 to 50	2	1	50
51 to 60	6	0	0
Above 60	5	0	0
Total	13	1	7.69

**Duration of the surgical procedure**: Analysis of incidence of SSI in relation to duration of the operative procedure showed an increased incidence in patients with operative time more than 1.5 hours, with a mean of 10% compared to a mean value of 1.66% in those patients with operative time less than 1.5 hours, as shown in Table 4. By other means, 60% of the infections in our study occurred when the operative duration exceeded 2 hours.

**Table 4:** Duration of hospital stay and incidence of SSI.

Duration (Hourrs)	Total No. of patients	No. of patients infected	Percentage
<1	22	0	0
1–1.5	38	1	2.85
1.5–2	29	1	3.44
>2	11	3	27.27

**Time of occurrence of infection**: As shows in Table 5, analysis of incidence of infection in relation to the time of occurrence of infection postoperatively showed that 1 patient developed signs of SSI on the 3rd day after surgery and 2 patients each developed the same on the 6th and 12th day after the surgery.

**Table 5:** Incidence of infection in relation to the time of occurrence of infection.

Time of occurrence of infection in the postoperative period	No of patients infected
3 days	1
6 days	2
12 days	2
3 months	0
6 months	0
1 year	0

**Fig. 1:**
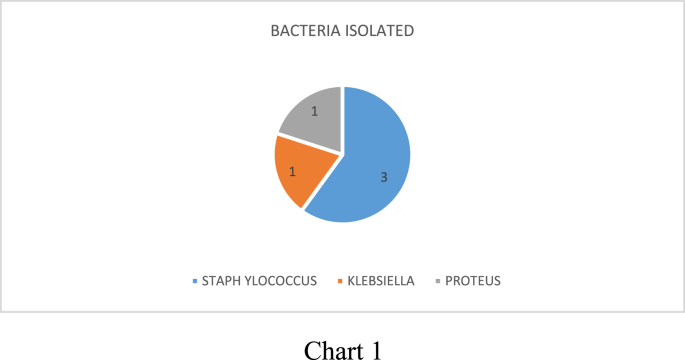
Pathogens that caused SSI.

**Pathogenic organisms:** Out of the 5 patients who developed SSI, 3 patients showed a positive wound swab culture for *Staphylococcus aureus* (60%) which included 2 cases of Methicillin Resistant Staph Aureus (MRSA) and 1 case of non- MRSA, 1 case showed *Klebsiella pneumoniae* (20%) and the other showed *Proteus vulgaris* (20%), as shown in Fig. 1.

## 4 Discussion

Surgical site infections (SSI), especially orthopaedic device related infections (ODRI), are associated with a high burden to the patients as well as the healthcare system, and remains an unresolved problem [[1], [2], [3], [4]]. ODRI are difficult to treat as they often result in the formation of “biofilm” over implants, and are often impossible to eradicate the infection without removal of the implants [12]. In ODRI, the life-long recurrence rate of infection is around 10–20%, particularly with Methicillin Resistant Staphylococcus Aureus (MRSA) [13,17]. These infections tend to be inevitable though meticulous precautions before, during and after surgery are being taken by the surgeons and healthcare workers.

Out of the total 100 patients included in our study, the incidence of SSI was 5%, which is comparable to that of Khan et al. who reported 5.68% in a similar study on 104 patients at a teaching hospital in Abottabad (Pakistan) [17]. In their study, advanced age, prolonged surgical time, history of smoking and the presence of skin abrasion at fracture sites were the associated risk factors for ODRI although statistical significance could not be proven. Another researcher, Ibtesam et al. from an Egyptian university, reported an incidence of ODRI of 6.28% in their study including 121 patients. In their study, a univariate analysis performed showed that ODRI was significantly associated with diabetes mellitus, age more than 50 years, American Society of Anaesthesiologist (ASA) score greater than 2, duration of surgery more than 2 hours and the usage of drains [18].

In our study, the mean incidence of SSI was 8.82% in patients with age more than 50 years as compared to 3.03% in patients younger than 50 years. This result corresponds to the results of Ibtesam et al., who also showed a higher incidence of SSI in age more than 50 years in their study involving 158 patients. Similar results were shown by the study performed by Khan et al. who showed that age greater than 60 is an important risk factor associated with ODRI. The higher incidence of infection with increasing age could be attributed to multiple factors like low healing rate, malnutrition, malabsorption, increased catabolic process and low immunity [19,20]. Regarding the incidence of SSI in relation to sex, the incidence was 4.93% in males and 5.26% in females, showing no preponderance to either sex; this was again in correspondence to the study by Ibtesam et al.

The incidence of postoperative infection in relation to the duration of preoperative hospital stay had a mean incidence of 6.85% among patients who stayed more than 10 days, whereas the incidence was only 1.66% for those who stayed less than or equal to 10 days in our study. This could be because longer preoperative hospital stay may result in colonization with microorganisms resistant to various antimicrobials. These findings correlate with a previous study by Patel S et al. about SSI in various surgical procedures in a tertiary care hospital of western India. In his study, SSI was found to be 33% (4 out of 12 patients) in patients with a preoperative hospital stay of 7–13 days [13].

Regarding diabetic patients, the incidence of infection in our study was found to be 7.69% (1 out of 13 diabetic patients). Patel et al., in his study on ODRI showed that out of the 22 patients with diabetes that were included, 36.4% (8 patients) incurred SSI compared to only 13.5% in those without diabetes (24 patients out of 178). Moreover, the National Academy of Science reported a higher SSI rate in diabetic patients which supports the findings of our study [21]. Moreover, a Univariate analysis on the incidence of SSI conducted by Ibtesam et al. on 121 patients showed that SSI in their study was significantly associated with diabetes mellitus, with a P value 0.009.

In our study, the mean incidence of postoperative infection in relation to the duration of operative procedure was 10% in those cases where the duration of surgery exceeded 1.5 hours (4 out of 50 patients), and only 1.66% in those with the duration of procedure less than 1.5 hours (1 out of 60). These findings were consistent with that of other workers [[13], [22], [23], [24]]. Patel et al., in his study on 200 surgical patients, found that those with an operative duration more than the 75th percentile had a higher chance of SSI. This could be due to prolonged exposure of the tissues to the surrounding environment with increased air-borne contamination.

In our study, out of the five infected patients, *Staphylococcus aureus* was the predominant organism causing SSI. Among the three cases of *Staphylococcus aureus* infection, two were MRSA. Similar results were seen by Jadrajka et al., Ibtesan et al. and Vishal et al., who noted *Staphylococcus aureus* as the predominant pathogen causing SSI in their studies [18,25,26]. Also, it has been shown that about 15–30% of the healthy population carry *Staphylococcus aureus* in their nares which could opportunistically cause SSI [27]. In addition, fomites, which include bedsheets have also been proven to be the reservoir of *Staphylococcus aureus* [28]. Another interesting finding noted in our study was that in one of the patients with MRSA, there was a chronic non healing ulcer remote to the operative site which also harboured the same organism. This finding is comparable to the study by Garibaldi et al., who showed an infection rate of 16% in patients with remote infection compared to 6.1% in patients without remote infection [29]. All the isolated organisms in our study showed resistance to common antibiotics used for prophylaxis, and showed variable sensitivity towards aminoglycosides and tetracyclines.

Regarding the management of infected cases in our study, one patient required only repeated dressing and appropriate antibiotics, three patients required wound wash and debridement, and one patient required flap cover procedure.

## 5 Conclusion

The findings of our study show that SSI are apparently inevitable in spite of taking all the standard aseptic precautions. The risk factors associated with SSI were advanced age, presence of diabetes, increased duration of preoperative hospital stay more than 10 days and prolonged duration of surgery more than 1.5 hours. Thus, reducing the duration of preoperative hospital stay, reducing the duration of the surgical procedure as far as possible are the controllable factors that can reduce the incidence of SSI while maintaining all the standard aseptic precautions.

## Sources of funding

There were no sources of funding received for the current study.

## Provenance and peer review

Not commissioned, externally peer-reviewed.

## Conflicts of interest

None.

## Ethical approval

Yes. IRB GRH0055420.

## Consent

Yes, patient consent was obtained.

## Author contribution

1. Niranj Ganeshan Radhamony-study concept, data collection, data analysis, writing the paper
2. Radhu Raj- data analysis, statistics, writing the paper
3. Sivakumar Raju- study design, supervision, review

## Registration of research studies

1. Name of the registry: www.researchregistry.com
2. Unique Identifying number or registration ID: researchregistry7216
3. Hyperlink to your specific registration: https://www.researchregistry.com/browse-the-registry#home/

## Guarantor

Niranj Ganeshan Radhamony, 18 Sharman Close, Stoke on Trent, UK niranj.gr@gmail.com.

## Declaration of competing interest

The authors do not have any conflicts of interest to disclose.
